# Final results of a phase II study of nivolumab in Japanese patients with relapsed or refractory classical Hodgkin lymphoma

**DOI:** 10.1093/jjco/hyaa117

**Published:** 2020-08-08

**Authors:** Dai Maruyama, Yasuhito Terui, Kazuhito Yamamoto, Noriko Fukuhara, Ilseung Choi, Junya Kuroda, Kiyoshi Ando, Akira Hattori, Kensei Tobinai

**Affiliations:** 1 Department of Hematology, National Cancer Center Hospital, Tokyo, Japan; 2 Department of Hematology and Oncology, Cancer Institute Hospital of JFCR, Tokyo, Japan; 3 Department of Hematology and Cell Therapy, Aichi Cancer Center, Nagoya, Japan; 4 Department of Hematology/Rheumatology, Tohoku University Graduate School of Medicine, Sendai, Japan; 5 Department of Hematology, NHO Kyushu Cancer Center, Fukuoka, Japan; 6 Division of Hematology and Oncology, Department of Medicine, Kyoto Prefectural University of Medicine, Kyoto, Japan; 7 Department of Hematology and Oncology, Tokai University, Isehara, Japan; 8 Medical Affairs, Ono Pharmaceutical Co., Ltd., Osaka, Japan

**Keywords:** nivolumab, Hodgkin lymphoma, survival, disease progression, interstitial lung disease

## Abstract

**Background:**

Many patients with classical Hodgkin lymphoma show increased programmed death-1 ligand expression in Reed–Sternberg cells. We report the final results of a phase II study of nivolumab, an anti-programmed death-1 monoclonal antibody, in Japanese patients with relapsed or refractory classical Hodgkin lymphoma.

**Methods:**

Japanese patients with previously treated classical Hodgkin lymphoma (aged ≥ 20 years) were administered nivolumab (3 mg/kg on Day 1 of 14-day cycles) until progressive disease, an unacceptable adverse event, or another clinically relevant reason. Treatment could continue beyond progressive disease at the investigator’s discretion in selected patients.

**Results:**

Seventeen patients (median age: 63.0 years) were enrolled. The median follow-up was 38.8 months. One patient with non-Hodgkin lymphoma was excluded from efficacy analyses. The centrally assessed overall response rate in 16 classical Hodgkin lymphoma patients was 87.5% (95% confidence interval = 61.7–98.4%) and the disease control rate was 93.8% (95% confidence interval = 69.8–99.8%). The median (95% confidence interval) duration of response and progression-free survival were 8.5 (2.4–12.6) and 11.7 (1.8–42.3) months, respectively. The 3-year overall survival rate was 80.4% (95% confidence interval = 50.6–93.2%). Nivolumab was continued beyond progressive disease in seven patients; six were alive at the data cut-off. Adverse drug reactions occurred in all 17 patients with grades 3–4 adverse drug reactions in eight patients and no grade 5 adverse drug reactions. Pulmonary toxicities occurred in five patients; four of these occurred ≥17 months after starting nivolumab.

**Conclusion:**

Nivolumab is effective and tolerable in Japanese relapsed or refractory classical Hodgkin lymphoma patients. Continued monitoring may be necessary to detect late-onset pulmonary toxicities.

**Clinical trial registration:**

JapicCTI-142755 (Japan Pharmaceutical Information Center).

## Teaser

We report the final results of a phase II study of nivolumab in Japanese patients with classical Hodgkin lymphoma. Nivolumab was effective with reductions in tumor size in responders and was tolerable.

## Introduction

Classical Hodgkin lymphoma (cHL) is a relatively rare form of lymphoma that is less common in Japan than in the USA and Europe (1). The treatment of early-stage cHL is based on chemotherapy (doxorubicin, bleomycin, vinblastine and dacarbazine) plus radiotherapy, while patients with advanced cHL may receive chemotherapy with or without radiotherapy (2,3). These treatments achieved a complete remission (CR) rate of 92% among patients with early cHL and 82% among patients with advanced cHL (3). However, ~20% of patients experience relapse or refractory disease and require autologous stem cell transplantation (ASCT) or alternative treatments. The prognosis after ASCT failure is typically poor, especially once the cytotoxic chemotherapies have been exhausted.

Brentuximab vedotin (BV), the first antibody–drug conjugate to be approved for relapsed HL, is now widely used as part of frontline therapy (4,5). Recent studies have revealed that BV could be administered in combination with chemotherapy in previously untreated patients, offering a small increase in progression-free survival (PFS) compared with chemotherapy alone (6). Nevertheless, alternative treatments are still needed for patients who experience disease relapse following ASCT or disease progression after BV and/or chemotherapy.

Programmed death-1 (PD-1) ligand expression in Reed–Sternberg cells is increased in many patients due to genomic alterations in 9p24.1, and it is now acknowledged that PD-1 ligand overexpression contributes to an immunosuppressive microenvironment and poor prognosis of cHL (7,8). Therefore, blocking this pathway might offer an alternative treatment strategy for cHL. Nivolumab and pembrolizumab are anti-PD-1 monoclonal antibodies that block the signal preventing activated T cells from targeting cancer cells and thereby act as immune checkpoint inhibitors (9,10). Owing to the increased expression of PD-1 in cHL, several studies examined the effects of nivolumab in patients with relapsed cHL following ASCT [e.g. (11,12)], and it is now approved for the treatment of relapsed or progressive cHL.

CheckMate 205, a pivotal phase II study, revealed that nivolumab was associated with good clinical responses in three cohorts of patients, namely BV-naïve patients, patients who received BV after ASCT, and patients who received BV before and/or after ASCT (13,14). Similar results were reported in the KEYNOTE-087 study in which pembrolizumab was administered in three groups of patients with lymphoma progression after ASCT and subsequent BV, salvage chemotherapy and BV ineligible for ASCT, and ASCT without post-transplantation BV (15,16).

Here, we describe the final results of a Japanese phase II study (ONO-4538-15) that assessed the efficacy and safety of nivolumab in patients with relapsed or refractory cHL. The results of this study were previously reported at a cut-off date of 16 March 2016 (17), at which time the 6-month PFS and overall survival (OS) rates were 60.0% and 100.0%, respectively, with a median follow-up of 9.8 months. However, median PFS and OS had not been reached, as 12 patients were still on treatment at the cut-off. Of note, our study included older patients with a median age of 63.0 years compared with a median of 34 years in CheckMate 205 (13,14), and there are limited long-term efficacy or safety data for nivolumab in older patients with cHL. Therefore, a longer follow-up of patients in the Japanese phase II study was necessary; here, we report the final data from this study.

## Materials and methods

### Ethics

This study conformed with the Declaration of Helsinki, Good Clinical Practice and Japanese regulations, was approved by the institutional review board at each site, and was registered on a public database (Japan Pharmaceutical Information Center, ID: JapicCTI-142755). We obtained written informed consent from each patient before enrollment. The final analyses reported here were embedded in the study protocol approved by the institutional review boards and the consent documents signed by the patients at enrollment.

### Patients

As previously described (17), patients with a histopathological diagnosis of cHL aged ≥ 20 years were eligible if they met the criteria for prior ASCT and BV therapy (prior ASCT, contraindicated for ASCT or refusal to undergo ASCT; prior BV therapy or patients who were clinically unqualified for BV, even if they had not received it). Patients with nodular lymphocyte-predominant Hodgkin lymphoma, central nervous system involvement, concurrent or history of chronic autoimmune disease, a current or past history of interstitial lung disease or pulmonary fibrosis, history of organ allograft or allogeneic hematopoietic stem cell transplantation, or prior treatment with therapeutic antibodies or pharmacotherapies to regulate T cells were excluded. Additional inclusion and exclusion criteria are described in our prior report (17).

### Study design and treatments

Nivolumab was administered on Day 1 of each 14-day cycle at a dose of 3 mg/kg and was to be continued until progressive disease (PD), an unacceptable adverse event (AE), or another clinically relevant reason. Nivolumab could be continued beyond investigator-assessed progression if protocol-predefined criteria were met, including stable performance status, and there was a perceived clinical benefit. Patients treated beyond initial PD were required to discontinue in the event of further progression (further increase in tumor burden of ≥10%). Computed tomography or magnetic resonance imaging was to be performed in cycles 4, 8, 12, 18, 24, 32, 40, 48 and 61, and every 13 cycles thereafter, while fluorodeoxyglucose positron emission tomography was to be performed on Day 15 in cycles 8, 12 and 24. Diagnostic imaging was to be continued until the next treatment for cHL or confirmation of PD/relapse, if possible (ideally every 8–12 weeks), in patients who discontinued nivolumab for a reason other than PD and if their response at the time of discontinuation was classified as CR, partial remission (PR) or stable disease (SD).

The Revised International Working Group criteria for malignant lymphoma (18) were used to assess the responses to nivolumab. The primary endpoint was the centrally assessed objective response rate (ORR). The secondary efficacy and safety endpoints are described in detail in our prior report (17). Efficacy endpoints included the investigator-assessed ORR, disease control rate (DCR), time to and duration of response, PFS, OS, B symptoms and target lesion size. Lesion size was defined as the sum of the products of the longest diameters of the target lesion. For safety endpoints, the Common Terminology Criteria for Adverse Events version 4.0 was used to classify AEs. We defined adverse drug reactions (ADRs) as AEs for which a relationship with the study drug could not be denied. For the purpose of this study, we focused on several ADRs of special interest namely endocrine disorders, adrenal disorders, diabetes mellitus, pituitary disorders, thyroid disorders, gastrointestinal toxicity, hepatotoxicity, pulmonary toxicity, renal toxicity, skin toxicity and hypersensitivity/infusion reaction. The investigators were to collect information on ADRs of special interest for up to 28 days after the final dose of the investigational drug.

PD ligand-1 (PD-L1) expression and 9p24.1 alterations were determined by double immunohistochemistry and fluorescence *in situ* hybridization on formalin-fixed paraffin-embedded tumor tissue samples, as previously described (7).

### Statistical analyses

As we previously described, it was planned to enroll 15 patients (17). Efficacy outcomes were assessed using the efficacy analysis set, which comprised all patients with cHL who received at least one dose of nivolumab. Safety outcomes were assessed using the safety analysis set, which comprised all patients who received at least one dose of nivolumab. The Clopper–Pearson method was used to determine the 95% confidence interval (CI) for ORR. The Kaplan–Meier method was used to estimate PFS and OS. Prespecified analyses of response rates, PFS and OS were performed for the following subgroups of patients: best response to prior BV and age (<65 and ≥65 years). All data analyses were done using SAS version 9.3 (SAS Institute, Cary, NC, USA).

## Results

### Patients

The first patient was enrolled on 18 March 2015, and the final observation date for the last patient was 22 November 2018. A total of 17 patients were enrolled and treated with nivolumab, with a median follow-up of 38.8 months (safety analysis set). The median follow-up of 16 patients with confirmed cHL was 38.3 months (full analysis set). The other patient was diagnosed with B-cell lymphoma, unclassifiable. This patient was subsequently excluded from the efficacy analysis set but was included in the safety analysis set (17). The characteristics of the 17 patients are summarized in (Table 1). There were 13 males and four females, with a median age of 63.0 years (range 29–83 years), and a median time since diagnosis of 24.0 months. The median number of prior chemotherapy regimens was three. Prior therapies included BV in all 17 patients, ASCT in five patients, and radiotherapy in nine patients.

**Table 1:** Patient characteristics. Reprinted from (17)

		Value
Sex, *n* (%)	Male	13 (76.5)
	Female	4 (23.5)
Age, *n* (%)	Median (range)	63.0 (29–83)
	<65 years	9 (52.9)
	≥65 years	8 (47.1)
Time since diagnosis (months)	Median (range)	24.0 (8.9–89.0)
ECOG PS, *n* (%)	0	8 (47.1)
	1	9 (52.9)
Disease subtype, *n* (%)	Nodular sclerosis	8 (47.1)
	Lymphocyte-rich	0
	Mixed cellularity	6 (35.3)
	Lymphocyte-depleted	2 (11.8)
	Unclassified	1 (5.9)
Disease stage at study enrollment, *n* (%)	II	4 (23.5)
	III	5 (29.4)
	IV	8 (47.1)
B symptoms, *n* (%)	Absent	12 (70.6)
	Present	5 (29.4)
PD-L1 expression^a^, *n* (%)	Positive (≥1%)	11 (100.0)
	Negative (<1%)	0
	Could not be determined	0
9p24.1 status, *n* (%)	Polysomy	0
	Copy gain	4 (50.0)
	Amplification	4 (50.0)
Relapse or refractory (to most recent therapy)^b^, *n* (%)	Relapse	1 (5.9)
	Refractory	16 (94.1)
Number of prior chemotherapy regimens	Median (range)	3 (2–5)
Prior BV therapy		17 (100.0)
BOR to BV, *n* (%)	CR	2 (11.8)
	PR	5 (29.4)
	SD	4 (23.5)
	PD	5 (29.4)
	NE	1 (5.9)
Prior ASCT, *n* (%)		5 (29.4)
BOR to ASCT, *n* (%)	CR	3 (60.0)
	PR	1 (20.0)
	SD	1 (20.0)
Prior radiotherapy, *n* (%)		9 (52.9)

ASCT, autologous stem cell transplantation; BOR, best overall response; BV, brentuximab vedotin; CR, complete remission; ECOG PS, Eastern Cooperative Oncology Group performance status; NE, not evaluable; PD, progressive disease; PD-L1, programmed death ligand-1; PR, partial remission and SD, stable disease.

^a^Only patients with available specimens were counted.

^b^Relapse indicates best response of CR to the most recent prior therapy, and refractory indicates best response of PR, SD or PD to the most recent prior therapy.

Nivolumab was administered for a median of 24 cycles (range 4–77). The median relative dose intensity was 88.8% (range 70.2–99.2%).

### Tumor responses and survival

Table 2 shows the best responses, as assessed by the central review committee and by the investigators, in the 16 patients with cHL. The centrally assessed ORR was 87.5% (95% CI = 61.7–98.4%). The lower bound exceeded the prespecified threshold of 20%. CR and PR were achieved in five and nine patients, respectively. The DCR, defined as CR + PR + SD, was 93.8% (95% CI = 69.8–99.8%). We previously reported that the response could not be evaluated in one patient (17). In the final analysis, this patient was classified as CR. The investigator-assessed ORR was 62.5% (95% CI = 35.4–84.8%), with CR in three patients, PR in seven patients and SD in three patients (Table 2).

**Table 2:** Best responses to nivolumab (efficacy analysis set, *n* = 16)

Assessment	Central review	Investigator review
ORR (CR + PR), *n* (%) [95% CI]	14 (87.5) [61.7–98.4%]	10 (62.5) [35.4–84.8%]
DCR (CR + PR + SD), *n* (%) [95% CI]	15 (93.8) [69.8–99.8%]	13 (81.3) [54.4–96.0%]
BOR, *n* (%)		
CR	5 (31.3)	3 (18.8)
PR	9 (56.3)	7 (43.8)
SD	1 (6.3)	3 (18.8)
PD	1 (6.3)	3 (18.8)
NE	0	0

BOR, best overall response; CI, confidence interval; CR, complete remission; DCR, disease control rate; NE, not evaluable; ORR, objective response rate; PD, progressive disease; PR, partial remission and SD, stable disease.

**Figure 1.**
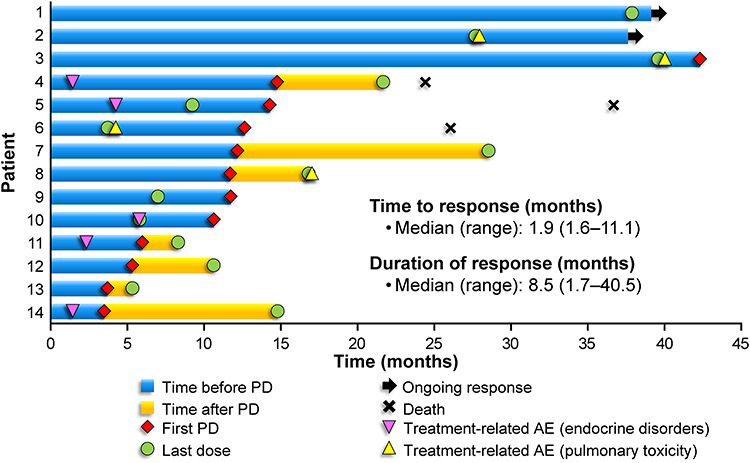
Treatment exposure and duration of response among 14 patients with partial or complete response. Central review according to the revised response criteria for malignant lymphoma (18). AE, adverse event and PD, progressive disease.

**Figure 2.**
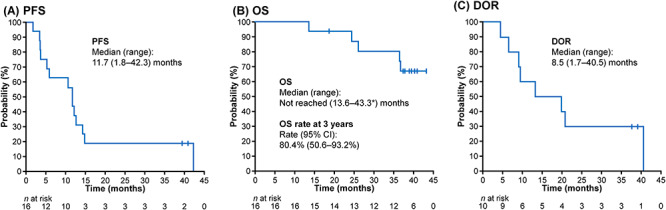
Kaplan–Meier plots of PFS (A), OS (B) and DOR (C) over a median follow-up of 38.3 months. Efficacy analysis set (*n* = 16). ^*^Censored value. CI, confidence interval; DOR, duration of response; OS, overall survival and PFS, progression-free survival.

Figure 1 shows the duration of response relative to treatment exposure in individual patients. The median time to response was 1.9 months after starting treatment, and the median duration of response was 8.5 months (95% CI = 2.4–12.6 months). The median PFS was 11.7 months (range 1.8–42.3 months; Fig. 2A). The 3-year OS rate was 80.4% (95% CI = 50.6–93.2%), and the median OS was not reached (Fig. 2B).

### Tumor dimensions

**Figure 3.**
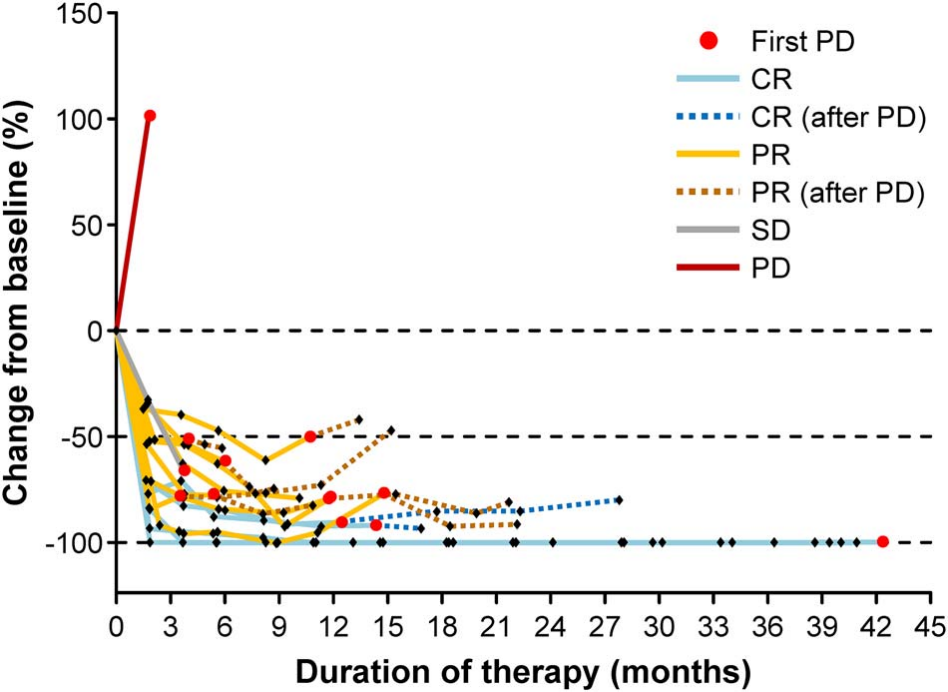
Spider plot for the change in target lesion size from baseline. Lesion size was calculated as the sum of the products of the diameters of the target lesion. Efficacy analysis set (*n* = 16). CR, complete remission; PD, progressive disease; PR, partial remission and SD, stable disease.

The target lesion size shrank by ~50–100% in 15 patients with a best response of SD or better (Fig. S1). These changes were evident by the first imaging assessment in cycle 4 (~8–10 weeks after starting therapy). These reductions in target lesion size were maintained through to study discontinuation/last follow-up in these 15 patients (Fig. 3). One patient with PD showed a large increase in target lesion size, potentially hyper-PD, at the time of the first imaging assessment in cycle 4 (Day 54) and nivolumab was discontinued. This patient was given chemotherapy, but no information was available on the type of chemotherapy or its outcomes other than survival; this patient died on Day 415. The prior treatments (disease response) in this patient were ABVD as first-line therapy (PR), CHASE as the second-line therapy (ineligible for autologous transplantation due to SD), BV as third-line therapy (PR followed by PD 2 months later) and nivolumab as fourth-line therapy.

### Tumor responses, OS and PFS in patient subgroups

Eight patients were considered to be resistant to prior BV, defined as a best overall response of SD, PD or not evaluable while on therapy. The ORR after starting nivolumab in these patients was 100% (95% CI = 63.1–100.0%), with CR in four patients and PR in four patients (Table 3). Median PFS in this subgroup was 13.3 months (range 3.7–42.3 months); the median OS was not reached.

**Table 3:** Best responses (central assessment) to nivolumab according to best response to prior BV administration and age at enrollment

Endpoint	Best response to prior BV^a^		Age at enrollment	
	CR or PR (*n* = 7)	SD, PD or NE (*n* = 8)	<65 years (*n* = 8)	≥65 years (*n* = 8)
ORR (CR + PR), *n* (%) [95% CI]	5 (71.4) [29.0–96.3%]	8 (100.0) [63.1–100.0%]	7 (87.5) [47.3–99.7%]	7 (87.5) [47.3–99.7%]
DCR (CR + PR + SD), *n* (%)	6 (75.0)	8 (100.0)	7 (87.5)	8 (100.0)
BOR, *n* (%)				
CR	1 (14.3)	4 (50.0)	3 (37.5)	2 (25.0)
PR	4 (57.1)	4 (50.0)	4 (50.0)	5 (62.5)
SD	1 (14.3)	0	0	1 (12.5)
PD	1 (14.3)	0	1 (12.5)	0
NE	0	0	0	0
Median DOR (range), months	4.2 (1.7–37.6^b^)	10.3 (1.7–40.5)	ND	ND
Median PFS (range), months	6.0 (1.8–39.4^b^)	13.3 (3.7–42.3)	11.7 (1.8–42.3)	11.4 (3.6–14.3)
Median OS (range), months	n/r	n/r	ND	ND

BOR, best overall response; BV, brentuximab vedotin; CI, confidence interval; CR, complete remission; DCR, disease control rate; DOR, duration of response; ND, not determined; NE, not evaluable; n/r, not reached; ORR, objective response rate; OS, overall survival; PD, progressive disease; PFS, progression-free survival; PR, partial remission and SD, stable disease.

^a^The response to prior BV was not evaluated in one patient.

^b^Censored value.

The 16 patients with cHL were also divided into two age groups, with eight patients aged <65 years and eight aged ≥65 years at enrollment. The best response of CR or PR was achieved in seven patients (87.5%) in both subgroups, with CR in three and two patients aged <65 and ≥65 years, respectively (Table 3). The median PFS was similar in both subgroups, being 11.7 months (range 1.8–42.3 months) and 11.4 months (range 3.6–14.8 months), respectively.

Among 10 patients positive for PD-L1 (≥1%), CR or PR was observed in nine with an ORR of 90.0% (95% CI = 55.5–99.7%). We also assessed responses according to the patient’s 9p24.1 status, defined as copy gain or amplification. The ORR was 100.0% (95% CI = 29.2–100.0%) in three patients with copy gain and 100.0% (95% CI = 39.8–100.0%) in four patients with amplification.

### Treatment beyond progression

**Figure 4.**
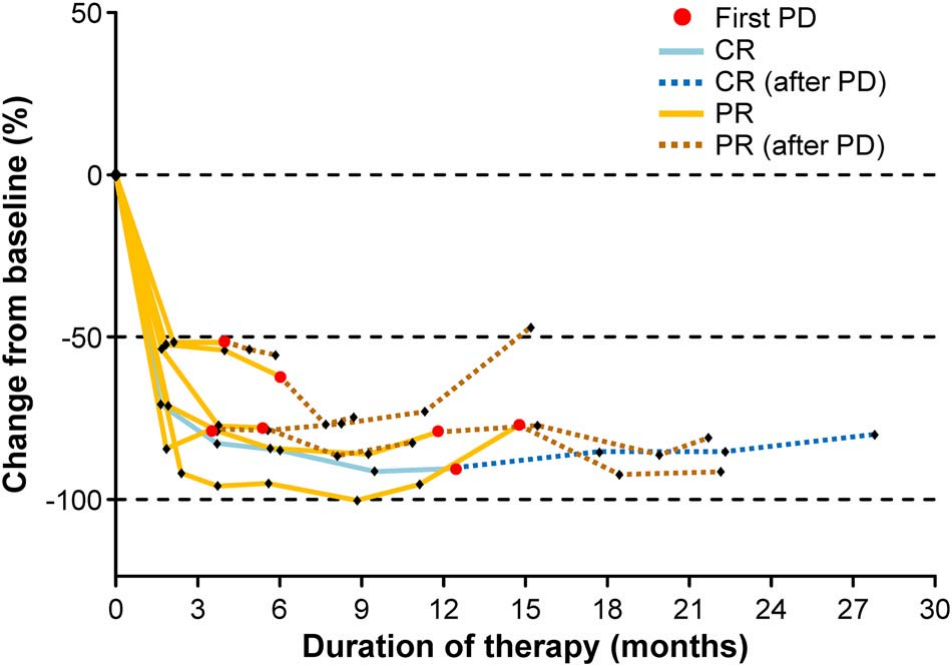
Spider plot for change in target lesion size from baseline for patients who continued nivolumab beyond PD. Lesion size was calculated as the sum of the products of the diameters of the target lesion. CR, complete remission; PD, progressive disease and PR, partial remission.

Nivolumab was continued beyond PD in seven patients as an investigational drug. PD was assessed by central review, and was due to an increased tumor diameter in three patients, the occurrence of a new lesion in three patients, and both factors in one patient. A spider plot for the seven patients who were treated beyond progression is provided in Figure 4. The response assessed prior to the initial PD (CR in one patient, PR in six patients) was maintained for several months to years after the initial PD until the patient’s last follow-up visit/death. In all seven patients, B symptoms had disappeared at the time of PD. Performance status (PS) was 0 in five patients and 1 in two patients. Of these seven patients treated beyond progression, one died on Day 743 (PS was 0 at the time of PD in this patient), and the other six patients were alive at the data cut-off.

### Subsequent treatments

Twelve patients received subsequent therapies after PD, including BV (three patients), gemcitabine + dexamethasone + CDDP (two patients), gemcitabine (two patients), ifosfamide + CBDCA + etoposide (two patients) and dexamethasone + CDDP + cytarabine (one patient). In addition, three patients received nivolumab following its approval in Japan in actual clinical practice. These three patients did not include any of the patients who received nivolumab beyond PD described in the previous section.

### Safety

The safety of nivolumab was assessed in all 17 patients (safety analysis set). There were no deaths related to AEs or ADRs. AEs and ADRs occurred in all 17 patients (Table 4). Fourteen ADRs in eight patients (47.1%) were classified as grades 3–4. There were no grade 5 ADRs. The most common ADRs were pyrexia, rash and pruritus. No new ADRs were identified during the longer follow-up that had not been described in our previous report (17). Five patients (29.4%) discontinued treatment because of ADRs. These ADRs were interstitial lung disease in two patients and increased amylase, peripheral neuropathy and rash in one patient each. All of these were classified as grades 3–4, except for one case of interstitial lung disease (grade 2). Serious ADRs occurred in six patients (35.3%) and included serious grades 3–4 ADRs in four patients (23.5%) (Table 4). All serious ADRs resolved, except for fulminant type 1 diabetes in one patient (19). The administration of nivolumab was temporarily withdrawn with a delay in subsequent doses due to an ADR in seven patients. The grades 3–4 ADRs that led to the temporary withdrawal of nivolumab were lymphopenia, thrombocytopenia, proctitis, fever, liver dysfunction, pneumonia, lipase increased, hyperglycemia and hyponatremia. Pulmonary toxicities occurred in five patients, with a grade ≥ 3 event in one of these patients. Two patients discontinued nivolumab due to pulmonary toxicities. The pulmonary toxicities were interstitial lung disease in three patients and pneumonitis in two patients. The pulmonary toxicities occurred at 4.2, 17.0, 27.9 and 40.0 months after starting nivolumab in patients with cHL and at 33.8 months in the patient with non-Hodgkin lymphoma. Other grade ≥ 3 ADRs of special interest included endocrine disorder, diabetes mellitus and skin toxicity in one patient each. The majority of ADRs occurred within 216 days from the start of treatment, although some patients experienced late-onset ADRs (≥216 days after starting treatment), with rash, interstitial lung disease, increased amylase, abnormal hepatic function, pneumonitis and increased alkaline phosphatase in two patients each and muscle spasm in one patient.

**Table 4:** Adverse drug reactions (safety analysis set, *n* = 17)

	Grade, *n* (%)		Time from the start of treatment, *n* (any grade)^a^^,^^b^	
	Any grade	Grades 3–4	<216 days	≥216 days
Overall ADRs	17 (100.0)	8 (47.1)		
ADRs in ≥2 patients				
Pyrexia	7 (41.2)	1 (5.9)	7	0
Rash	7 (41.2)	1 (5.9)	5	2
Pruritus	6 (35.3)	0	6	0
Hypothyroidism	5 (29.4)	0	5	0
Fatigue	3 (17.6)	0	3	0
Interstitial lung disease	3 (17.6)	1 (5.9)	1	2
Increased amylase	2 (11.8)	1 (5.9)	0	2
Abnormal hepatic function	2 (11.8)	1 (5.9)	0	2
Hyponatremia	2 (11.8)	1 (5.9)	2	0
Malaise	2 (11.8)	0	2	0
Muscle spasm	2 (11.8)	0	1	1
Myalgia	2 (11.8)	0	2	0
Pneumonitis	2 (11.8)	0	0	2
Increased blood alkaline phosphatase	2 (11.8)	0	0	2
Serious ADRs	6 (35.3)	4 (23.5)		
Interstitial lung disease	2 (11.8)	1 (5.9)		
Proctitis	1 (5.9)	1 (5.9)		
Pyrexia	1 (5.9)	1 (5.9)		
Abnormal hepatic function	1 (5.9)	1 (5.9)		
Hyponatremia	1 (5.9)	1 (5.9)		
Fulminant type 1 diabetes mellitus	1 (5.9)	1 (5.9)		
Pneumonitis	1 (5.9)	0		
Rash	1 (5.9)	1 (5.9)		
ADRs of special interest				
Endocrine disorders	6 (35.3)	1 (5.9)		
Fulminant type 1 diabetes mellitus	1 (5.9)	1 (5.9)		
Hypothyroidism	5 (29.4)	0		
Gastrointestinal toxicity	2 (11.8)	0		
Diarrhea	1 (5.9)	0		
Enteritis	1 (5.9)	0		
Hepatotoxicity	3 (17.6)	0		
Increased γ-glutamyl transferase	1 (5.9)	0		
Liver function test abnormality	1 (5.9)	0		
Increased blood alkaline phosphatase	2 (11.8)	0		
Pulmonary toxicity	5 (29.4)	1 (5.9)		
Interstitial lung disease	3 (17.6)	1 (5.9)		
Pneumonitis	2 (11.8)	0		
Skin toxicity	9 (52.9)	0		
Pruritus	6 (35.3)	0		
Rash	7 (41.2)	1 (5.9)		
Hypersensitivity/infusion reaction	1 (5.9)	0		
Infusion reaction	1 (5.9)	0		

Some patients experienced multiple ADRs.

^a^216 days was the longest-available cut-off value.

^b^Time of onset data by grades 3–4 ADRs were not available.

ADR, adverse drug reaction.

## Discussion

This Japanese phase II study was performed to determine the long-term efficacy and safety of nivolumab for the treatment of relapsed or refractory cHL after multiple lines of therapy, including BV. A key finding of this long-term study is that nivolumab was successfully continued beyond PD in seven patients, of which six were alive at the database cut-off. In addition, we also observed the possibility of late-onset pulmonary toxicities, occurring as long as 40 months after the start of nivolumab. The final results also demonstrate the clinical responses to nivolumab in terms of the ORR (87.5%), DCR (93.8%), median DOR (8.5 months), PFS (11.7 months) and a high long-term survival rate of 80.4% at 3 years. These results provide confirmation of the earlier results of this study at a median follow-up of 9.8 months (17). The results are also generally consistent with those reported in the international CheckMate 205 study (13,14) in which the ORR was 69% in the overall cohort, the median duration of response was 16.6 months and the median PFS was 14.7 months.

Treatment beyond PD is an emerging treatment paradigm that allows clinicians to extend treatment with immunotherapies following PD, providing the patients satisfy appropriate clinical criteria. This concept has been applied to melanoma (20,21) and may also be appropriate in cHL, as demonstrated in CheckMate 205, in which nivolumab was continued beyond PD in 70 of 105 patients (13). In our study, nivolumab could have been continued after the first confirmation of PD or if the patient had not received a dose of nivolumab within the last 6 weeks for specific reasons or ≥6 weeks in the case of steroid tapering after the treatment of drug-related AE. Seven patients continued nivolumab in our study, all of whom were in good condition, with PS of 0 or 1 and with the disappearance of B symptoms. Notably, none of these patients experienced serious immune reaction-related AEs during treatment with nivolumab. In CheckMate 205, the criteria included stable PS, and the patient was deemed to experience a clinical benefit of continuing treatment; patients were to discontinue nivolumab in the event of further progression (≥10% further increase in tumor burden) (13). Although our study included a small number of patients, our findings nevertheless support the emerging concept of treatment beyond PD.

Other key efficacy outcomes relate to the median age of patients (63.0 years) and the long duration of follow-up (38.3 months). Both of these factors exceed those of other phase II studies of patients with relapsed or refractory cHL (18 months in CheckMate 205 and 27.6 months in KEYNOTE-087) (13,16). Despite the older age of patients in our study, the ORR was still comparable with that in CheckMate 205, although we must consider that the smaller sample size might introduce some bias. We also found no difference in the efficacy of nivolumab between patients aged <65 and ≥65 years with ORR of 87.5% in both subgroups and median PFS of 11.7 and 11.4 months, respectively.

Drug-induced interstitial lung disease is thought to account for 3–5% of all cases of interstitial lung disease (22). A notable finding of our study was the emergence of late-onset pulmonary toxicities, which occurred up to 40.0 months after the start of nivolumab. To our knowledge, this is the first study to report late-onset pulmonary toxicities in HL patients treated with an immune checkpoint inhibitor. Interstitial lung disease and pneumonitis (any grade) occurred in 17.6 and 11.8% of patients respectively in the present study, while pneumonitis occurred in 2.1% of patients in CheckMate 205 (13) and 4.8% of patients in KEYNOTE-087 (16). The higher incidence of pulmonary toxicities in this study appears to be driven by the late events, which may be related to the relatively long follow-up of our study. A pulmonary toxicity occurred in one patient who continued treatment beyond PD (at 17.9 months after starting nivolumab), which suggests that careful, ongoing monitoring is also essential in all patients. Nevertheless, only two patients discontinued treatment due to pulmonary toxicities, and only one event was classified as grade ≥ 3.

In terms of other safety findings, the overall safety profile was consistent with that described in our prior report (17). Furthermore, there were no new previously undescribed ADRs, and there was no marked increase in the frequency of ADRs between the prior cut-off date (16 March 2016) and the final observation date (22 November 2018). Despite the relatively small number of patients enrolled in the study, the duration of exposure was relatively long, which allowed us to detect events that might take some time to develop.

Finally, we wish to discuss some possible limitations, including the relatively small sample size, which means the study was underpowered for comparisons among subgroups of patients. However, this was unavoidable owing to the rarity of the disease and the indication, limiting the size of the patient population. Longer follow-up may also be necessary to assess OS in patients treated with nivolumab for cHL. Studies in real-world settings are also needed to confirm the use of nivolumab in actual clinical practice.

In conclusion, the results of this study show that in this cohort of patients with a poor prognosis due to their older age, the treatment outcomes (i.e. ORR, DCR, PFS and OS) were generally comparable with those obtained in a study of younger patients conducted overseas (13). Our study also involved a longer follow-up compared with the majority of prior studies of relapsed or refractory cHL. As a consequence, we detected several late-onset pulmonary toxicities that necessitate careful, long-term monitoring of patients on nivolumab. Finally, our study suggests that nivolumab could be continued beyond PD in selected patients with good clinical status (good PS and disappearance of B symptoms), none of whom developed severe immune reaction-related AEs during the long-term follow-up, although these findings warrant further verification.

## Supplementary Material

Figure_S1_hyaa117

## Abbreviations

ADR: adverse drug reaction
AE: adverse event
ASCT: autologous stem cell transplantation
BV: brentuximab vedotin
CI: confidence interval
CR: complete remission
DCR: disease control rate
ORR: objective response rate
OS: overall survival
PD: progressive disease
PFS: progression-free survival
PR: partial remission
PS: performance status
SD: stable disease

## References

[1] Bray F, Ferlay J, Soerjomataram I, Siegel RL, Torre LA, Jemal A Global cancer statistics 2018: GLOBOCAN estimates of incidence and mortality worldwide for 36 cancers in 185 countries. CA Cancer J Clin 2018;68:394–424. 30207593 10.3322/caac.21492

[2] Ansell SM Hodgkin lymphoma: diagnosis and treatment. Mayo Clin Proc 2015;90:1574–83. 26541251 10.1016/j.mayocp.2015.07.005

[3] Makita S, Maruyama D, Maeshima AM, et al. Clinical features and outcomes of 139 Japanese patients with Hodgkin lymphoma. Int J Hematol 2016;104:236–44. 27086350 10.1007/s12185-016-2007-1

[4] Allen PB, Winter JN Controversies in the approach to initial therapy of Hodgkin lymphoma. Curr Oncol Rep 2019;21:39. 30919161 10.1007/s11912-019-0788-0

[5] Alperovich A, Younes A Targeting CD30 using brentuximab vedotin in the treatment of Hodgkin lymphoma. Cancer J 2016;22:23–6. 26841013 10.1097/PPO.0000000000000168 PMC5042201

[6] Connors JM, Jurczak W, Straus DJ, et al. Brentuximab vedotin with chemotherapy for stage III or IV Hodgkin’s lymphoma. N Engl J Med 2018;378:331–44. 29224502 10.1056/NEJMoa1708984 PMC5819601

[7] Roemer MG, Advani RH, Ligon AH, et al. PD-L1 and PD-L2 genetic alterations define classical Hodgkin lymphoma and predict outcome. J Clin Oncol 2016;34:2690–7. 27069084 10.1200/JCO.2016.66.4482 PMC5019753

[8] Roemer MGM, Redd RA, Cader FZ, et al. Major histocompatibility complex class II and programmed death ligand 1 expression predict outcome after programmed death 1 blockade in classic Hodgkin lymphoma. J Clin Oncol 2018;36:942–50. 29394125 10.1200/JCO.2017.77.3994 PMC5877802

[9] Ansell SM Nivolumab in the treatment of Hodgkin lymphoma. Clin Cancer Res 2017;23:1623–6. 27881581 10.1158/1078-0432.CCR-16-1387

[10] Ansell SM Immunotherapy of Hodgkin lymphoma: mobilizing the patient’s immune response. Cancer J 2018;24:249–53. 30247261 10.1097/PPO.0000000000000331

[11] Ansell SM, Lesokhin AM, Borrello I, et al. PD-1 blockade with nivolumab in relapsed or refractory Hodgkin’s lymphoma. N Engl J Med 2015;372:311–9. 25482239 10.1056/NEJMoa1411087 PMC4348009

[12] Yared JA, Hardy N, Singh Z, et al. Major clinical response to nivolumab in relapsed/refractory Hodgkin lymphoma after allogeneic stem cell transplantation. Bone Marrow Transplant 2016;51:850–2. 26828905 10.1038/bmt.2015.346

[13] Armand P, Engert A, Younes A, et al. Nivolumab for relapsed/refractory classic Hodgkin lymphoma after failure of autologous hematopoietic cell transplantation: extended follow-up of the multicohort single-arm phase II CheckMate 205 trial. J Clin Oncol 2018;36:1428–39. 29584546 10.1200/JCO.2017.76.0793 PMC6075855

[14] Younes A, Santoro A, Shipp M, et al. Nivolumab for classical Hodgkin’s lymphoma after failure of both autologous stem-cell transplantation and brentuximab vedotin: a multicentre, multicohort, single-arm phase 2 trial. Lancet Oncol 2016;17:1283–94. 27451390 10.1016/S1470-2045(16)30167-X PMC5541855

[15] Chen R, Zinzani PL, Fanale MA, et al. Phase II study of the efficacy and safety of pembrolizumab for relapsed/refractory classic Hodgkin lymphoma. J Clin Oncol 2017;35:2125–32. 28441111 10.1200/JCO.2016.72.1316 PMC5791843

[16] Chen R, Zinzani PL, Lee HJ, et al. Pembrolizumab in relapsed or refractory Hodgkin lymphoma: two-year follow-up of KEYNOTE-087. Blood 2019;134:1144–53. 31409671 10.1182/blood.2019000324 PMC6776792

[17] Maruyama D, Hatake K, Kinoshita T, et al. Multicenter phase II study of nivolumab in Japanese patients with relapsed or refractory classical Hodgkin lymphoma. Cancer Sci 2017;108:1007–12. 28267244 10.1111/cas.13230 PMC5448600

[18] Cheson BD, Pfistner B, Juweid ME, et al. Revised response criteria for malignant lymphoma. J Clin Oncol 2007;25:579–86. 17242396 10.1200/JCO.2006.09.2403

[19] Munakata W, Ohashi K, Yamauchi N, Tobinai K Fulminant type I diabetes mellitus associated with nivolumab in a patient with relapsed classical Hodgkin lymphoma. Int J Hematol 2017;105:383–6. 27696192 10.1007/s12185-016-2101-4

[20] Long GV, Weber JS, Larkin J, et al. Nivolumab for patients with advanced melanoma treated beyond progression: analysis of 2 phase 3 clinical trials. JAMA Oncol 2017;3:1511–9. 28662232 10.1001/jamaoncol.2017.1588 PMC5710191

[21] Beaver JA, Hazarika M, Mulkey F, et al. Patients with melanoma treated with an anti-PD-1 antibody beyond RECIST progression: a US Food and Drug Administration pooled analysis. Lancet Oncol 2018;19:229–39. 29361469 10.1016/S1470-2045(17)30846-X PMC5806609

[22] Skeoch S, Weatherley N, Swift AJ, et al. Drug-induced interstitial lung disease: a systematic review. J Clin Med 2018;7:356. doi: 10.3390/jcm7100356. PMC6209877 30326612

