# Supplementary material for: Final results of a phase II study of nivolumab in Japanese patients with relapsed or refractory classical Hodgkin lymphoma
Source: Jpn J Clin Oncol. 2020 Aug 8;50(11):1265–73. doi: 10.1093/jjco/hyaa117 (PMC7579338; doi:10.1093/jjco/hyaa117)

**Figure S1** Waterfall plot for best reduction from baseline in target lesion size

*CR* complete remission, *PD* progressive disease, *PR* partial remission


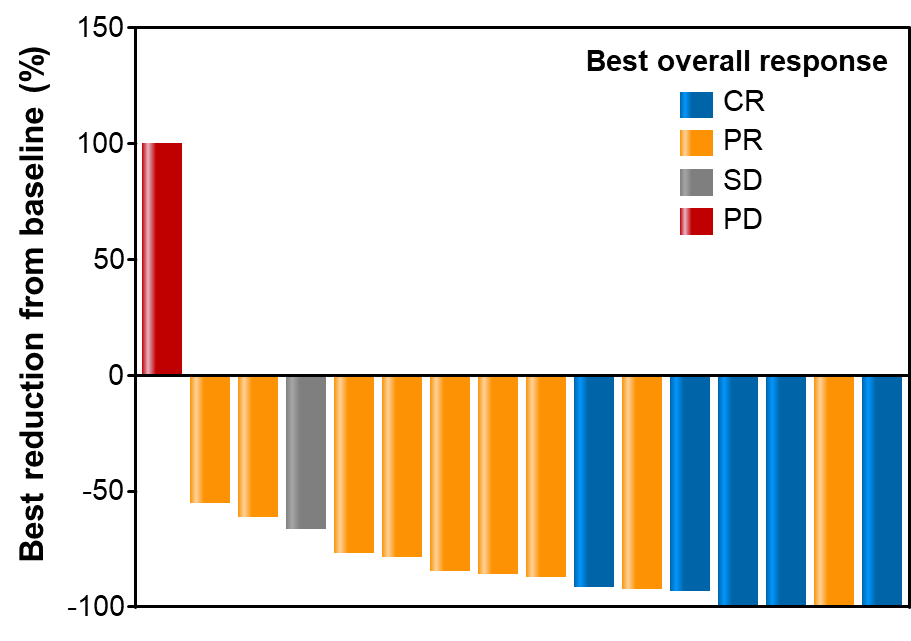

Supplement: Figure_S1_hyaa117 [file figure_s1_hyaa117.doc]
